# Understanding the abnormal thermal behavior of nanofluids through infrared thermography and thermo-physical characterization

**DOI:** 10.1038/s41598-021-84292-9

**Published:** 2021-03-01

**Authors:** Adela Svobodova-Sedlackova, Alejandro Calderón, Camila Barreneche, Pablo Gamallo, A. Inés Fernández

**Affiliations:** 1 Departament de Ciència de Materials i Química Física, Universitat de Barcelona, C/Martí i Franqués 1, 08028 Barcelona, Spain; 2 Institut de Química Teòrica i Computacional, IQTCUB, Universitat de Barcelona, C/Martí i Franqués 1, 08028 Barcelona, Spain

**Keywords:** Energy storage, Materials for energy and catalysis

## Abstract

Nanofluids (NFs) are colloidal suspensions of nanoparticles (NPs) within a base fluid. Unlike conventional mixtures, NFs exhibit dramatically enhanced properties, such as an abnormal increase in heat capacity at low concentration of NPs (e.g., C_p_ values 30% higher than the base material value). Understanding the thermo-physical behavior of NFs is essential for their application as thermal energy storage systems. In this study, we analyze a sodium nitrate ionic system containing 1 wt%, 3 wt% and 7 wt% of SiO_2_ NPs with different techniques like infrared thermography, infrared spectroscopy and differential scanning calorimetry (DSC) in order to shed light on the mechanism behind the increase of C_p_. The themographies reveal the presence of a colder layer on top of the NF with 1 wt% of NPs whereas this layer does not appear at higher concentrations of NPs. The IR spectrum of this foamy top layer evidences the high amount of SiO_2_ bonds suggesting the clustering of the NPs into this layer linked by the nitrate ions. The linking is enhanced by the presence of hydroxyls in the NPs’ surface (i.e., hydroxilated NPs) that once mixed in the NF suffer ionic exchange between OH^−^ and NO_3_^−^ species, leading to O_2_–Si–O–NO_2_ species at the interface where a thermal boundary resistance or Kapitza resistance appears (R_T_ = 2.2 m^2^ K kW^−1^). Moreover, the presence of an exothermic reactive processes in the calorimetry of the mixture with 1 wt% of NPs evidences a reactive process (ionic exchange). These factors contribute to the heat capacity increase and thus, they explain the anomalous behavior of the heat capacity in nanofluids.

## Introduction

Energy storage systems are key technologies for achieving the transition to renewable energies. Such systems allow to improve energy efficiency making renewable energy more viable^1^. An example of this is the great advances made in the efficiency of solar energy by using thermal energy storage (TES) systems. At present, more than 70% of concentrated solar power (CSP) projects have integrated TES systems^2^, and such power plants reached an installed capacity of 4.5 GW at the end of 2019^3^. Due to the importance of the storage systems for the viability of CSP technology, it is necessary to keep on improving them by increasing both their energy efficiency and their capacity of continuously generating electricity, without forgetting their competitiveness from an economic point of view.

Materials science and technology are essential to meet these improvements. Nanoscience has made great strides forward in the field of energy storage and retrieval. Nanofluids (NFs) are a promising option for a wide variety of techniques in the future, including TES systems^4,5^. NFs are a colloidal suspension of nanoparticles (NPs) within a base fluid. Unlike conventional mixtures of different components, NFs exhibit dramatically enhanced properties, such as an abnormal increase in heat capacity^6^ at low concentration of NPs (e.g., C_p_ values 30% higher than the base material value)^7^. According to this, the use of materials as NFs in TES systems allows increasing the CSP plant’s efficiency as well as reducing the costs associated to the thermal storage process. Precisely, the energy density of a material is the product of its specific heat, C_p_, and its density. Therefore, an increase in C_p_ of a TES material leads to a proportional increase in the energy density, which also means a decrease in the size of the storage tanks required, hence allowing more efficient and compact systems.

Despite the great potential of NFs, we are still far away from understanding fundamental questions concerning them as simply the NF stability^8^. Moreover, it does not help the discrepancies observed in experimental and simulated values of some thermo-physical properties (e.g., C_p_) of NFs already published^9,10^. Thus, there is no a clear explanation for the observed phenomena like the increase in heat capacity observed at low concentration of nanoparticles, although several hypotheses are available^11–13^. Literature suggests that Infrared Thermography (IRT) technique it is a very useful technique to study the thermal behavior of NFs and it has been successfully applied to the study of vaporization and wettability of NFs^14–16^. This work, therefore, tries to provide an explanation of the anomalous increase in C_p_ of sodium nitrate NFs containing one of the commonly used NPs: silicon dioxide. To this end, high-resolution IR is used as it allows observing the thermal behavior of NFs, together with Fourier-transform infrared spectroscopy (FT-IR) combined with differential scanning calorimetry (DSC), which allows to characterize the physico-chemical properties of the NFs.

### Infrared thermography and Kapitza resistance

Infrared thermography allows the study of heat transfer in NFs and their thermal behavior. The Stefan-Boltzmann law establishes that the total energy radiated by a material, E, is directly proportional to the fourth power of its temperature, via the Eq. (1):

1 $$\mathrm{E}=\upvarepsilon \cdot \upsigma \cdot {\mathrm{T}}_{\mathrm{e}}^{4}$$

where $\sigma =\mathrm{5,67}.{10}^{-8} \frac{W}{{m}^{2}\cdot {K}^{4}}$ is the Boltzmann constant of proportionality, ${T}_{e}$ is the effective temperature (absolute temperature of the surface) and $\varepsilon$ is the emissivity of the material.

**Figure 1:**
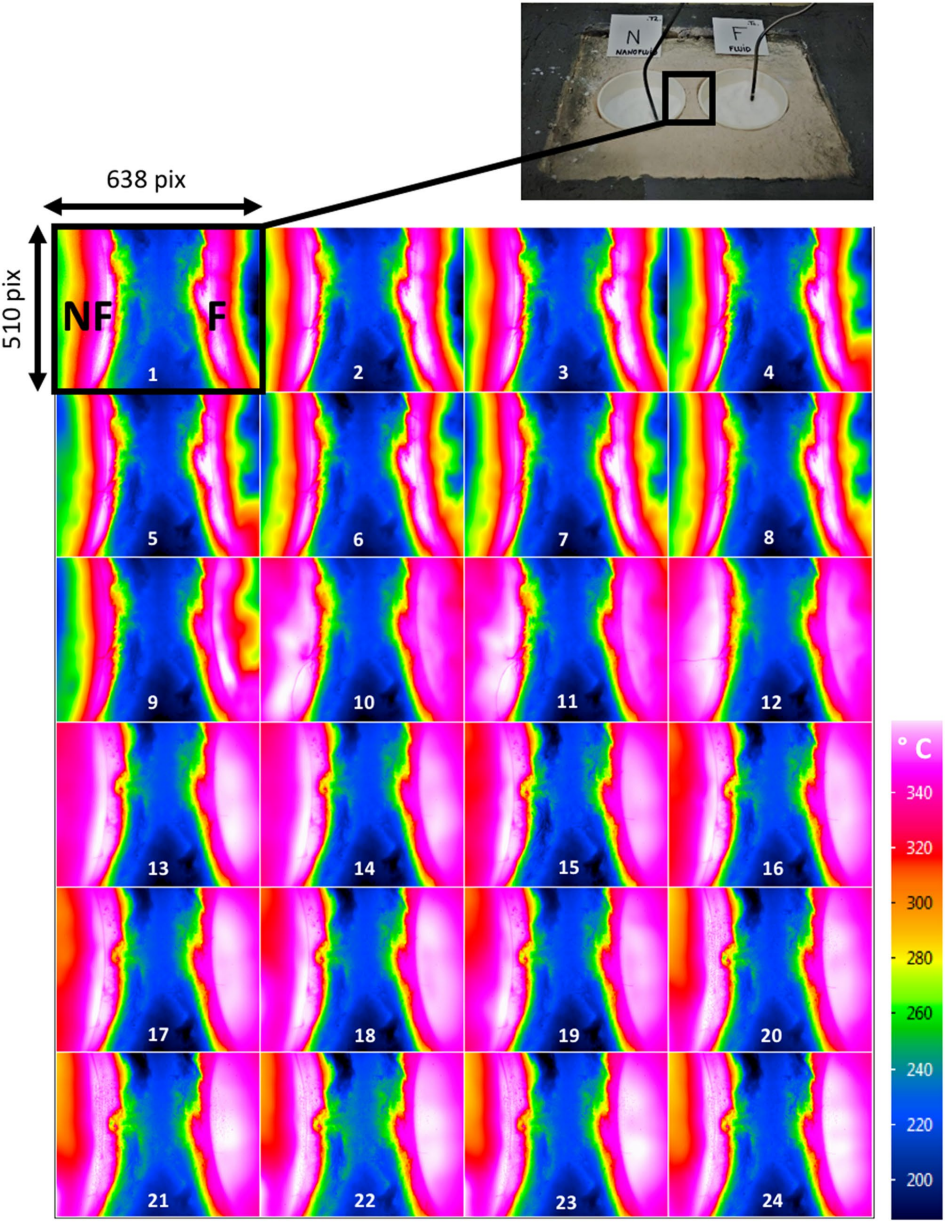
Infrared thermographies during the heating process of the nanofluid sample, NF (left, NaNO_3_ + 1 wt% SiO_2_) and the fluid sample F (right, NaNO_3_). The electrical power communicated to both samples is the same along the experiment.

The thermographies for the NF and the base fluid obtained from a high-resolution infrared camera are shown in Fig. 1, where different images (1–24) were taken at different times (every 15 min). For each image, the left-hand side corresponds to the NF (i.e., sodium nitrate with 1 wt% of SiO_2_) in the set-up and the right-hand side corresponds to the base fluid (i.e., pure sodium nitrate), represented as (F). Both samples are always subjected to the same heating by means of electrical resistances, so the temperature profile observed in each sample is related to the thermal behavior of each of them. Temperature of samples is controlled directly by thermocouples and indirectly, inferred from the thermographies although the maximum temperature that camera can measure is 340 °C. The experimental device used for obtaining the thermographies has been developed by the University of Barcelona (see Methodology section).

**Figure 2:**
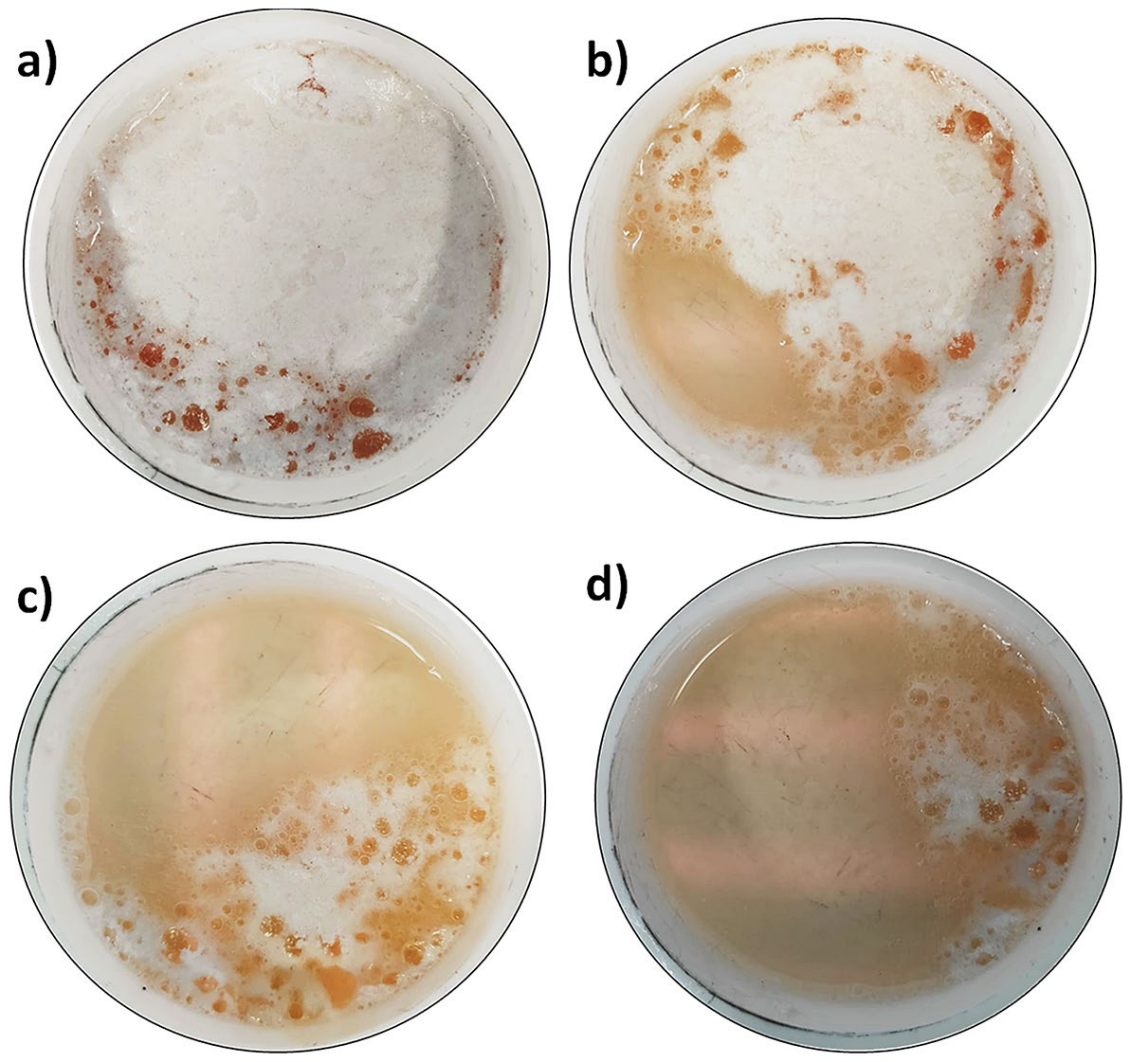
NF sample with 1 wt% of NPs at different temperatures, from 300 °C (**a**) to 450 °C (**d**). It is evident the formation of the foamy-like white semisolid layer on top of the sample that is colder than the liquid phase as it can be seen in Fig. 1, left hand side of images (15) to (24).

As it can be inferred from the thermographies, heating takes place in various stages:

- Stage 1: from images (2) to (8), a very similar temperature profile is observed for both samples, with the fluid (F) and the NF reaching approximately 292 °C ± 5 °C and 295 °C ± 6 °C, respectively, in (8).
- Stage 2: starting at image (9), the fluid (right) begins to melt (320 °C ± 4 °C), while the NF (left) is at a lower temperature (306 °C ± 4 °C) and still solid.
- Stage 3: from images (10) to (14), the phase transition is completed in both samples and therefore, at the end of this stage, both are in liquid phase with a similar temperature: approximately 355 °C ± 3 °C and 337 °C ± 10 °C for F and NF, respectively. It is worth noting that the NF has a less homogeneous temperature. The melting of the NF requires a higher temperature than the one for melting the pure salt. This behavior is just the opposite at what should be expected for a non-pure species according to classical theories^17^.
- Stage 4: from image (15) to (24) domains at lower temperature appear in the NF sample, despite the increase in heat flow. At this point, a foamy-like white layer appears on top of the NF sample (Fig. 2). The foamy-like white layer is formed from the beginning of the melting process (~ 308 °C) (Fig. 2a) and it persists up to approximately 450–500 °C, at which temperature the layer melts (Fig. 2d). The presence of this top foamy layer (i.e., new phase) corresponds with the lower temperature regions observed in the thermographs in Fig. 1, images (15) to (24), i.e., stage 4. Furthermore, the formation of the new phase generates additional interphases in the system (e.g., liquid–solid interphase) that produce thermal boundary resistance or Kapitza resistance (R_T_)^18,19^ (i.e., a sudden change in temperature between foamy top layer and the bulk fluid). It is possible to obtain the Kapitza resistance from the variation of temperature between the foamy top layer and the bulk liquid fluid (ΔT) and the heat flux crossing the interface (q) since R_T_ = ΔT/q. An accurate exploration of the thermographies in which the top layer is present (stage 4) show an average difference in temperature of 55.8 $\pm 3$ °C between the fluid and the foamy semisolid layer in the NF sample. According to the power of heat communicated to the samples (112.01 W) and the interface area between the top layer and the fluid (i.e., almost the crucible’s area 44.18 cm^2^), the resulting interfacial thermal resistance is R_T_ = 2.2 m^2^ K kW^−1^.

The anomalous behavior observed in the thermographs shown in Fig. 1 (variation of the melting parameters and a decrease in the thermal radiation from the surface as the thermal flow increases), and the evidence offered by Fig. 2 (formation of a semisolid foamy layer), indicate that in order to analyze and understand the abnormal behavior of NFs it is necessary to perform a detailed thermo-physical characterization of both the top foamy layer and the bulk fluid. The characterization has been focused on the NF sample with 1 wt% SiO_2_ NPs because at this concentration it is observed the presence of the semisolid top phase. Once the foamy layer is formed both phases are separated, cooled down to room temperature and then, characterized using FT-IR and DSC techniques.

### Physicochemical characterization of NFs

**Figure 3:**
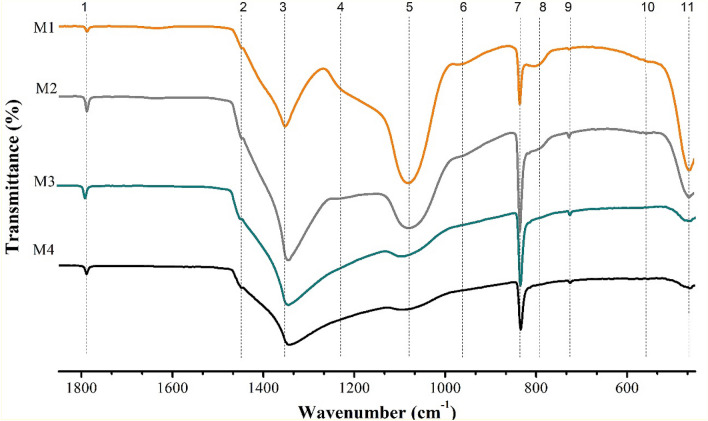
FT-IR spectra of the foamy semisolid top layer (M1, M2 and M3) along with the bulk melted NF with a 1 wt% concentration in NPs. M1 to M3 corresponds to the evolution of the top layer with temperature, from 300 to 450 °C. M4 corresponds to the spectrum of the entire NF once melted. The analyzed bands are identified by a number from 1 to 11. The 100% of transmittance for each spectrum is the base line at high wave numbers.

The FT-IR characterization allows to identify the principal functional groups present in the samples. Figure 3 shows the FT-IR spectra of the foamy semisolid layer when it is formed (M1) when it is heated (M2) and when it melts (M3). Moreover, M4 corresponds to the FT-IR spectrum of the entire NF once melted.

The analysis of the spectra has been done focusing on eleven bands (Fig. 3). The bands (1), (3) and (7) are characteristic of sodium nitrate and they are observed in all the series. Concretely, in M1 they correspond to the out-of-plane N=O bond bending (1789 cm^−1^), the symmetrical bending of NO_3_^−^ (1352 cm^−1^) and the asymmetric stretching of NO_3_^−^ (836 cm^−1^), respectively ^20,21^. It is notorious the slight blue shift of band (3) compared to the value for pure nitrate (1338 cm^−1^) and the red shift that it suffers as the top layer melts. Moreover, the width of a band is characteristic of the crystallinity of the material^22^, so the band is progressively narrowed from M4 to M1, indicating an increasing of crystallinity, with the greatest degree in M1, which corresponds to the formation of the foamy semisolid top layer. Moreover, band (7), the asymmetric NO_3_^−^ stretching mode, is also sensitive to the transition from the semisolid state (M1) to the liquid phase (M3 at 829 cm^−1^) with a slight red shift during the melting process^22^. Obviously, these facts are produced by an alteration in the force constant and the dipole moment of the NO_3_^−^ vibrational group, indicating that the sodium nitrate environment has been altered.

**Figure 4:**
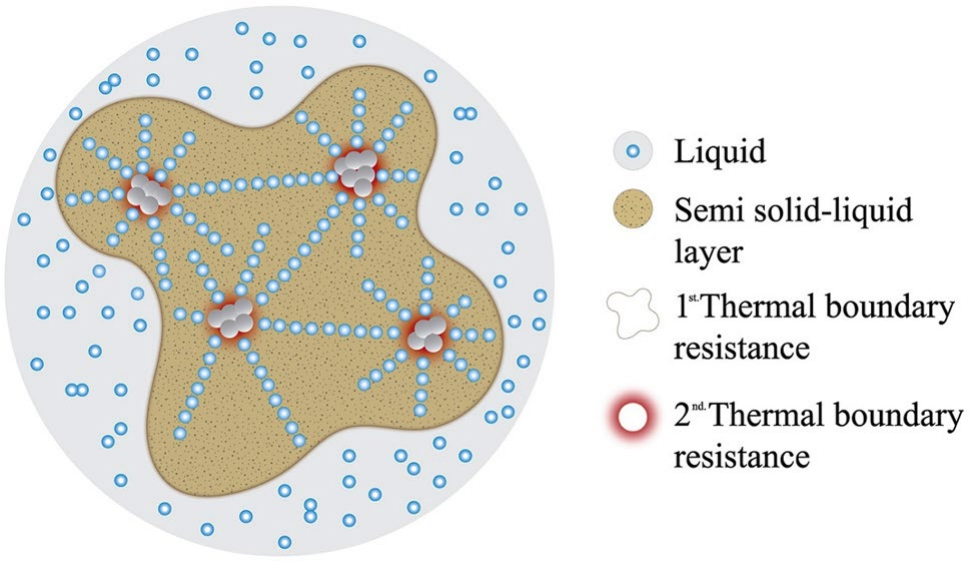
Schematic representation of nanofluid phases and interphases.

The characteristic bands of silicon dioxide have been also identified^23^. Band (5) is associated to the asymmetric stretching of SiO_x_ (1085 cm^−1^ for x = 2)^24^. In the samples, it is observed a broadening and a blue shift of band (5) from M1 (1081 cm^−1^) to M4 (1089 cm^−1^). Band (6) is the symmetric stretching of SiO_4_ only present in M1 (972 cm^−1^) and M2 (952 cm^−1^) and band (9) corresponds to the Si–O–Si symmetric stretching mode (724 cm^−1^)^25,26^ and band (11) corresponds to the symmetric bending mode of SiO_4_ (462 cm^−1^)^24,27^. Band (11) exhibits a rapid and considerable decrease from M1 (semisolid) to M4 (melting). According to Ref.^29^, the maximum intensity observed when the foamy top layer is formed owe to the adsorption of nitrate ions on the nanoparticle surface confirming an ionic exchange process between hydroxylated nanoparticles (O_2_–Si–OH) and sodium nitrate generating silica modified units like O_2_–Si–O–NO_2_ (i.e., O_2_–Si–NO_3_) and OH^−^ in the medium. This fact produces the formation of high specific-surface-area (SSA) silica layer modes that develop on the dissolving glass particles^25^ (see Fig. 4). It is worth noting that, taken together, the vibrational bands at 1385 cm^−1^, 870 cm^−1^ and 715 cm^−1^, which were weaker in M4, indicate the interfacial precipitation of crystalline Na^+^ cationic species^28^. Obviously, the new spatial ordering of the species causes the enhancement of the specific heat.

According to Ref.^28^, bands (3), (7) and (9) are associated to the low-frequency region by means of the so-called non-bridging oxygens (NBOs), due to such effects as vibrations of Si–O units, where the negative charge is electrostatically compensated by cationic species, in this case by Na^+^ cations^28^. This implies a lower reactivity. These bands disappeared during the melting of the surface layer (M3), implying there was diffusion of Na^+^ cations into the liquid^29^. Other additional bands were found: at 555 cm^−1^ (line 10), due to Si–O–Si asymmetric bending mode; while the signals at 815 cm^−1^ and 802 cm^−1^ (line 8), were associated with the O–Si–O bending mode for M1, and with Si–O–Si in the formed layer of SiO_2_^24^, in sample M2. Finally, in both samples, in the region of 1220 cm^−1^ (line 4), we identified the asymmetric stretching vibration of the surface component of Si–O–Si due to the formation of an interfacial SSA silica layer^17,19^.

### Calorimetric study

After confirming the ionic exchange between the fluid and the nanoparticles, a detailed thermophysical characterization is essential to study the thermal behavior of the samples.

**Figure 5:**
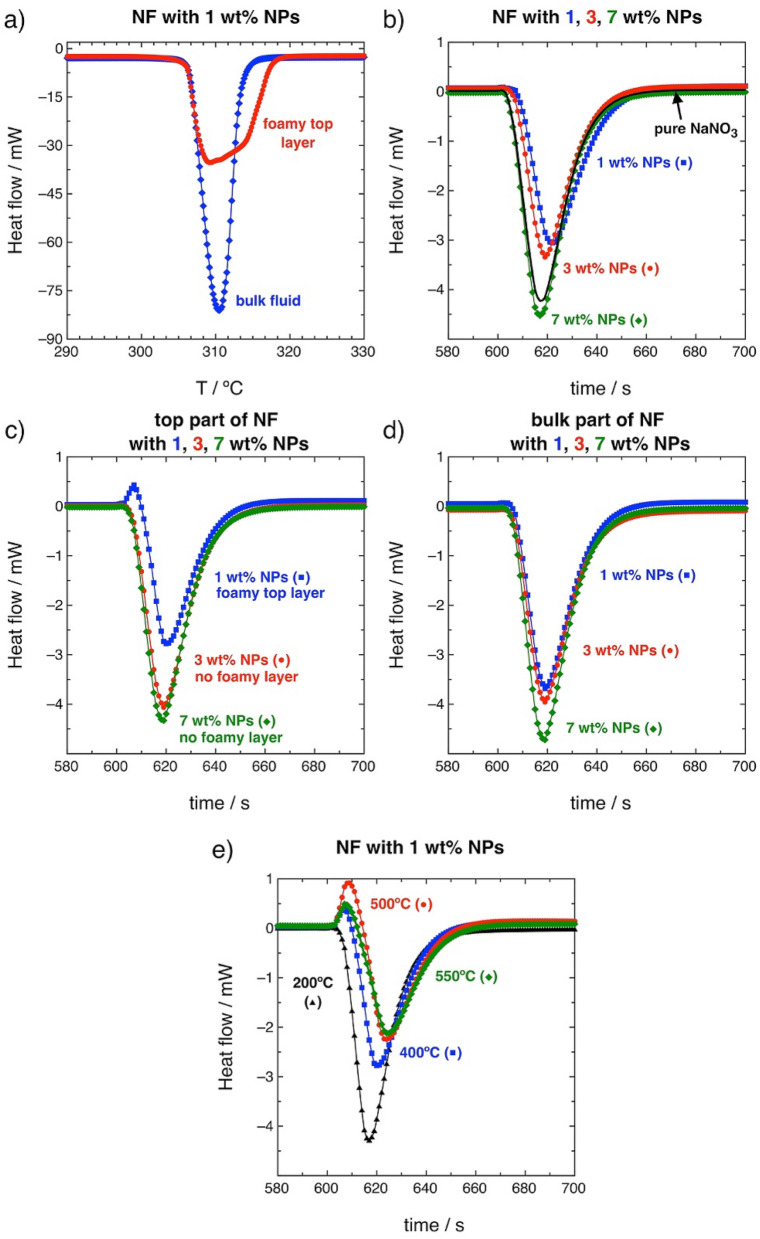
(**a**) Enthalpy of melting of the NF with 1 wt% of NPs: bulk fluid (blue rhombohedras) and foamy top layer (red circles). (**b**) Sensible heat at 400 °C for NFs with 1 wt% (blue squares), 3 wt% (red circles) and 7 wt% (green rhombohedras) of NPs along with the values for pure NaNO_3_ (black line). (**c**) Sensible heat at 400 °C of the top part of the NFs with 1 wt% (blue squares), 3 wt% (red circles) and 7 wt% (green rhombohedras) of NPs. The NF with 1 wt% of NPs is the only sample that exhibits the foamy top layer. (**d**) Sensible heat at 400 °C of the bulk fluid part of the NFs with 1 wt% (blue squares), 3 wt% (red circles) and 7 wt% (green rhombohedras) of NPs. (**e**) Temperature evolution of the foamy top layer’s sensible heat from 200 to 550 °C (NF with 1 wt% of NPs).

Figure 5a shows the heat flow absorbed as a function of temperature for the foamy top layer and for the bulk NF with a concentration of 1 wt% of NPs. The endothermic peak corresponds to the phase change (i.e., the enthalpy of melting). The top layer formed on the surface of the NF shows a broadening of the melting peak, compared to the bulk fluid sample. Consequently, the foamy top layer exhibits a greater enthalpy than the bulk fluid (i.e., − 180.4 J g^−1^ and − 154.5 J g^−1^, respectively). Therefore, there is an alteration of the thermal behavior between the phase formed at the surface and the rest of the system, consistent with the results determined using FT-IR.

The effect of the NPs’ concentration in the formation of the foamy top layer has been studied by means of isothermal measurements at 400 °C, to analyze the absorbed heat at this temperature. Figures 5b shows the heat flow absorbed (endothermic peaks) as a function of time at 400 °C for concentrations of NPs of 1, 3 and 7 wt%. The results show variations in the heat absorbed. At the maximum concentration of nanoparticles (7 wt%) the same behavior was observed as in the case of the base fluid without NPs. In contrast, as the concentration of NPs decreased, it took longer times for the system to reach thermal equilibrium compared to pure NaNO_3_. The isothermal analysis has been also done for the foamy top layer (Fig. 5c) and for the bulk fluid (Fig. 5d) of the NFs. However, the foamy top layer is only formed in the NF with 1 wt% of NPs although for NFs with 3 wt% and 7 wt% the analysis has been also done but for samples of the non foamy top part of the NF. As it can be inferred from Fig. 5c, the NF with 1 wt% of NPs is the only sample that showed an exothermic peak of 1.70 J g^−1^ in the isothermal calorimetry. This exothermic peak indicates the ionic exchange and reordering in the interphase NP and fluid that derives in the foamy semisolid top layer (Fig. 2). This top layer phase was not observed at higher NP concentrations as evidenced by the profiles observed in Fig. 5c. According to the literature, the increase in C_p_ depends strongly on the concentration of NPs reaching a maximum at around 1 wt%^31^ so it is straightforward that the increase of the C_p_ value comes from the foamy semisolid top layer. The nitrate-nitrite decomposition has been discarded for this peak since it is a process that occur in a tiny extent at 400 °C and to the fact that the peak only appears at 1 wt% of NPs and it should appear for all the samples.

The isothermal analysis of the foamy top layer has been done at different temperatures (Fig. 5e) showing that at temperatures higher than 500 °C the exothermal peak begins to decay. This temperature corresponds to the melting and blending of the foamy layer with the rest of the fluid.

The presence of the Si-NO_3_ bonds in the top layer increase the crystallinity of the system and consequently, the top layer melting point is higher than that of NaNO_3_. The measurement of the heat capacity has been carried out on the 1 wt% SiO_2_ NF as a whole and also split the system in two parts (i.e., the foamy top layer and the bulk fluid) at 350 °C. The C_p_ values obtained are 2.2 ± 0.1 J g^−1^ K^−1^ for the foamy top layer and 1.1 ± 0.1 J g^−1^ K^−1^ for the bulk fluid, really close to the value for pure NaNO_3_ (i.e., 1.2 ± 0.1 J g^−1^ K^−1^). The C_p_ value of the foamy top layer represents a 100 ± 10% of increasing with respect to the bulk phase. In the case of the C_p_ for the entire sample of NaNO_3_ + 1 wt% SiO_2_, a value of 1.3 ± 0.1 J g^−1^ K^−1^ was obtained, which represents an increase of the nanofluid value over the pure NaNO_3_ value of approximately 9.1 ± 0.9%. These findings also support the presence of Si–O–NO_2_ bonds that built the islands of NPs connected by sodium nitrate on the foamy top layer and the presence of simply NaNO_3_ in the bulk phase of the NF.

Given the coexistence of phases up to approximately 450 °C, we have calculated C_p_ for mixtures of the whole system using the calorimetric data. The law of mixtures can be expressed by the following Eq. (2):^17^

2 $${\mathrm{C}}_{\mathrm{p},\mathrm{ mixture}= \sum_{\mathrm{i}}{\mathrm{C}}_{{\mathrm{p}}_{\mathrm{i}}}\cdot {\mathrm{x}}_{\mathrm{i}}= {\mathrm{C}}_{{\mathrm{p}}_{\mathrm{i}}}\cdot \left(\frac{{\mathrm{n}}_{\mathrm{i}}}{{\mathrm{n}}_{\mathrm{t}}}\right)}$$

where for the *i*-species is defined the mole fraction (x_i_), the specific heat capacity (c_pi_) and the number of moles (n_i_) whereas n_t_ stands for the total number of moles of all the species in the system. In the case under study, the components of C_p_ are those formed by the foamy top layer (*tl*) and the bulk fluid (*bf*), Eq. (3):

3 $${\mathrm{C}}_{\mathrm{p},\mathrm{ mixture}= \sum_{\mathrm{i}}{\mathrm{C}}_{{\mathrm{p}}_{\mathrm{i}}}\cdot \left(\frac{{\mathrm{n}}_{\mathrm{i}}}{{\mathrm{n}}_{\mathrm{t}}}\right)={\mathrm{C}}_{{\mathrm{p}}_{\mathrm{tl}}}\cdot \left(\frac{{\mathrm{n}}_{\mathrm{tl}}}{{\mathrm{n}}_{\mathrm{t}}}\right)+{\mathrm{C}}_{{\mathrm{p}}_{\mathrm{bf}}}\cdot \left(\frac{{\mathrm{n}}_{\mathrm{bf}}}{{\mathrm{n}}_{\mathrm{t}}}\right)}$$

where: ${n}_{tl}=x\cdot n\left({SiO}_{2}\right)+y\cdot n\left({NaNO}_{3}\right); {n}_{bf}=\left(1-x\right)\cdot n\left({SiO}_{2}\right)+\left(1-y\right)\cdot n\left({NaNO}_{3}\right)$, with *x* being the fraction of SiO_2_ and *y*, that of NaNO_3_. By solving this equation, together with the law of conservation of mass and the constrain that the SiO_2_ is localized in the top layer (i.e., in the bulk fluid,$1-x\to 0$) the value of the molar mass of the foamy top layer ($\stackrel{-}{M}= \sum_{i}{M}_{i}\cdot {x}_{i}$) of approximately 83 g mol^−1^. From this, we can derive a relationship between density $\left({\rho }_{i}= \frac{{n}_{i}\cdot {M}_{i}}{{V}_{i}}\right)$, the molar mass $\left(\stackrel{-}{M}\right)$ and the volume of the top layer formed $\left({V}_{tl}\right)$: $V\propto 2\cdot {V}_{tl}$ and therefore: ${\rho }_{tl}\propto \frac{1}{2\cdot {V}_{tl}}\to {\rho }_{tl}\propto \frac{2}{V}$.

Therefore, the interphases formed in the system will be highly dependent on the amount of material to be analyzed. This dependence makes it difficult to carry out the measurements using conventional calorimetry equipment, since it uses small amounts of samples, of the order of 10–20 mg, which do not favor the formation of the surface layer or its identification due to the resolution of the equipment.

### Heat capacity: discussion

Heat capacity is a property that is well defined for gases and solids, but it lacks for a robust theory that describes the heat capacity of liquids^8,27^. It is therefore an extremely complex task to describe this property in NFs, where an extra difficulty comes from the presence of interphase effects.

Based on the classical view, NFs can be understood as fluids confined at the nano-scale^33^, in which a large number liquid-solid interfaces (LSIs) originate between the NPs surfaces and the fluid medium, where the heat transport properties become special and complex. When heat flows through the interphases between dissimilar media, a discontinuity in temperature occurs that is proportional to the thermal boundary resistance, or Kapitza resistance, due to the acoustic mismatch between the media in contact^34^, as observed in the thermographs we obtained (see Fig. 1). Therefore, at the interphases generated due to the formation of the foamy top layer, the phonon distribution function is disturbed by interfacial dispersion. One of the effects that this phenomenon provokes in measurements taken with calorimetry equipment is the variation in the thermal resistance of the material, therefore requiring longer times to reach thermal equilibrium, as can be observed in our calorimetric results. The concentration, size and dispersion of the NPs is of great importance for the formation of LSIs. This is because by reducing the system to the nanoscale, the effects of quantum confinement come into play. Such states are generated by nanostructured surfaces, causing a change in the occurrence of surface phonon modes^35^. Furthermore, liquid–solid interactions introduce stratification into the liquid near solid surfaces^8,31^. This is corroborated by the FT-IR results showing a higher degree of crystallization of the atoms in the liquid in the presence of NPs. The distribution of atoms in the liquid around solid surfaces is highly influenced by the nano-pattern formed by the distribution of NPs within the liquid. This distribution affects the adsorption characteristics of the surfaces, favoring—or not—the rearrangement of the liquid^19^. Thus, with a low concentration of NPs and high dispersion, (preventing agglomeration), a nano-pattern is generated which favors the formation of vibrational bands in surface modes (high SSA SiO_2_). This is dependent on the formation of the surface layer and the degree of liquid–solid coupling.

To understand the effect that these phenomena have on thermodynamic properties such as heat capacity, we can consider its increase as being attributed to the entropic contribution resulting from the multiple micro-regions of high SSA formed in the surface layer. These micro-regions cause an increase in the number of degrees of freedom of the system and in the vibrational entropy of the surfaces, which leads to an increase in C_p_. A schematic representation of the NF system is represented in Fig. 4. There are depicted the formation of the semi solid–liquid surface layer due to the atoms rearrangement around the nanoparticles surface and the two formed thermal boundaries: nanoparticles-surface layer-liquid.

## Conclusions

This study demonstrates experimentally for the first time the main phenomena involved in the abnormal increase of C_p_ in NFs. It provides a more detailed and unified vision and explanation of this phenomenon that has been studied considerably over recent years. Using infrared thermography, we established that there are regions which exhibit high temperature gradients. These gradients stem from the formation of LSIs with high-energy vibrational modes. The presence of these new interphases is due to exothermic processes linked to the reactivity between the ionic medium and the nanoparticulate oxide. The formation of LSIs directly influences the increase in the heat capacity of the system, increasing the number of degrees of freedom and the vibrational entropy of the surfaces. Furthermore, the dependency we found between the weight of sample analyzed and the volume of the surface layer that is formed can explain the large deviations between the published results of calorimetric values.

The results obtained in this study open the doors to the possibility of optimizing NFs, with the possibility of being able to design systems with the required C_p_ for different applications. In addition to radically changing the way we understand NFs, and specifically their applicability, this study also represents a step forward towards being able to scale them up.

## Materials and methods

### Materials

Sodium nitrate (Sigma Aldrich, 99.995%) and silicon oxide NPs between 5 and 15 nm in diameter (Sigma-Aldrich, 99.5%) were used. The NFs formulated for this study contained 1 wt%, 3 wt% and 7 wt% NPs by weight.

### Synthesis

To synthesize the materials the standard 1-step method was used, as it is the most straightforward and one of the most widely used for the synthesis of NFs^37^. The method consisted of: (1) preparation of 50 g of sample; (2) dissolution of this in 30 ml of distilled water; (3) 10 min of sonication for correct dispersion and homogenization of the NPs throughout the base material; (4) evaporation of the solvent in an oven at 105 °C, until all the water had evaporated and the material recrystallized; and finally, (5) extraction of the sample and milling in an agate mortar.

### Design and construction of the oven

#### Materials

Different materials were used to build the oven structure, including: a kaowool blanket, glass wool, refractory cement, refractory plates for resistances and high-temperature paint. For the electrical part we used a Kanthal A1 filament, a power supply, fans and five type K thermocouples. Samples were heated in sintered alumina crucibles.

#### Measurement system

**Figure 6:**
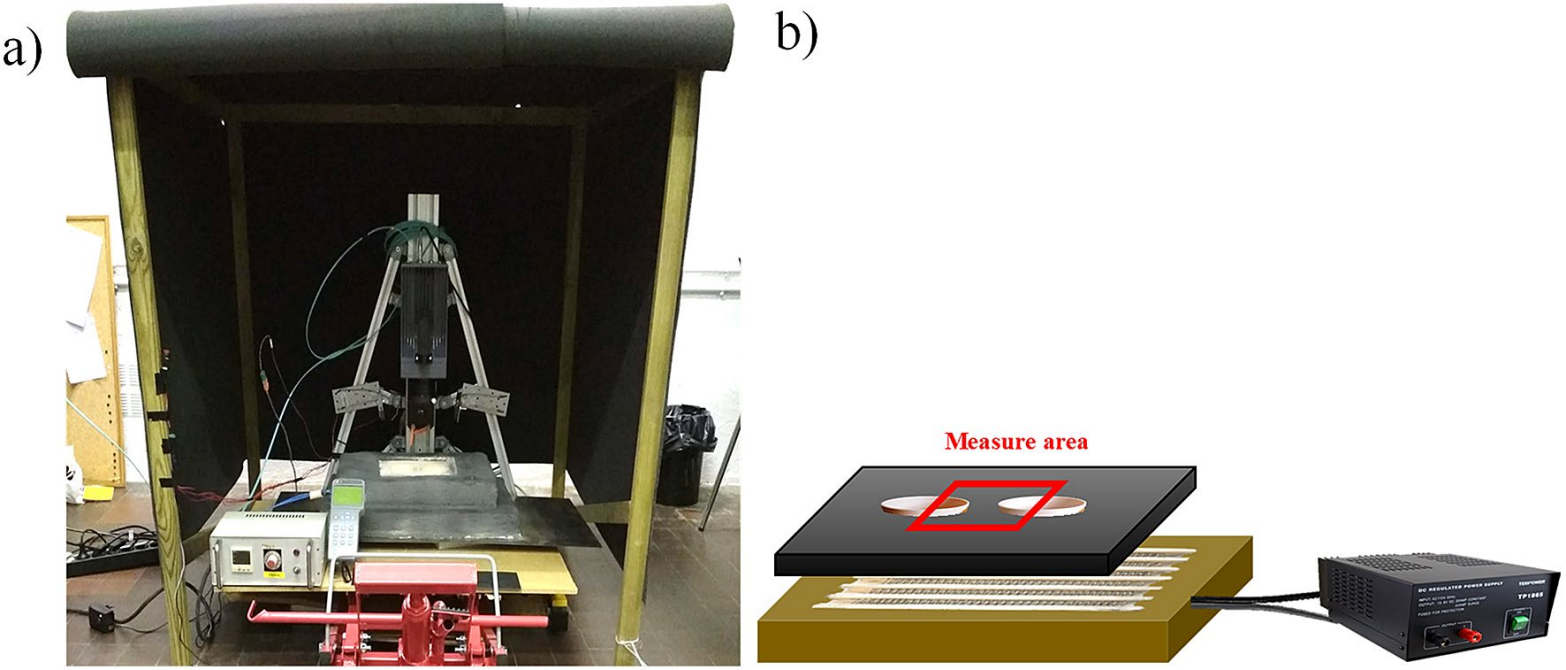
(**a**) Measurement system and (**b**) oven schematic representation and measurement area.

The complete measurement system is shown in Fig. 6. It consisted of the following parts: the infrared camera, the oven, the control and measurement system, and the temperature output. There was also a ventilation and safety system. Finally, a black box was used to isolate the system from its surroundings.

#### Infrared characterization

A high-resolution infrared camera (InfraTec GmbH) with IRBIS 3.1 professional software (www.infratec.eu) was used to obtain the infrared thermography images. The camera’s thermal resolution is 25 mK with an image resolution of 1,289 × 1,024 infrared pixels^2^. The area sampled was 510 × 638 pixels^2^ during 5.5 h per experiment. The thermographies were taken every 15 min lasting 40 s at a rate of 1,000 frames every 10 s (i.e., a total of 88,000 frames per experiment). The error associated to the temperature measure was obtained averaging 100 consecutive frames.

The first measurements were taken at room temperature (approximately 20 °C). Then, a heating gradient of 1 °C min^−1^ was applied, up to approximately 340 °C (maximum camera temperature).

#### Thermal characterization

The values of the heat capacity and enthalpy of the system were obtained at temperatures between 200 °C and 360 °C, with a differential scanning calorimeter (DSC 822e Star3 + , from Mettler Toledo). A constant flow of 50 mL min^−1^ of nitrogen was applied to preserve an inert atmosphere. The method described by Ferrer et al.^38^ was used to calculate C_p_. The amount of sample analyzed was 10 mg, performed in 100 µL aluminum crucibles. These crucibles were used for safety reasons (i.e., to prevent the fluid from rising up the walls of the crucible due to capillary action). Finally, each value was given as the mean of measurements from 20 independent samples (20 replicates) with the associated standard deviation.

#### Characterization of chemical structure and composition

FT-IR spectroscopy with attenuated total reflectance sampling, FT-IR ATR (Spectrum Two™, PerkinElmer) was used to determine the chemical composition of the samples. The instrumental error was 4 cm^−1^, and for each composition three independent samples were analyzed.
